# Effect of the β-tricalcium phosphate particle on dental enamel associated with 10% carbamide peroxide bleaching

**DOI:** 10.4317/jced.61300

**Published:** 2024-04-01

**Authors:** Marina-Paparotto Lopes, Iana-Maria-Costa Gonçalves, Julliana-Andrade da Silva, Danielle-Ferreira Sobral-Souza, Flávio-Henrique-Baggio Aguiar, Débora-Alves-Nunes-Leite Lima

**Affiliations:** 1 Undergraduate Student, Department of Restorative Dentistry, Piracicaba Dental School, University of Campinas – UNICAMP, P.O. BOX 52, 13414-903, Piracicaba, SP, Brazil; 2 DDS, MSc, Ph.D Student – Department of Restorative Dentistry, Piracicaba Dental School, University of Campinas – UNICAMP, P.O. BOX 52, 13414-903, Piracicaba, SP, Brazil; 3 DDS, MSc, Ph.D., Associate Professor – Department of Restorative Dentistry, Piracicaba Dental School, University of Campinas – UNICAMP, P.O. BOX 52, 13414-903, Piracicaba, SP, Brazil

## Abstract

**Background:**

Since bleaching gels can cause adverse effects on tooth enamel, it is important to evaluate new remineralizing agents on the market and their effects.

**Material and Methods:**

Seventy-five bovine enamel/dentin blocks (4x4x3mm) were randomly divided into six groups (n=10): Negative Control (NC) with no bleaching treatment or brushing; 10 CP (Carbamide Peroxide) (no brushing - Whiteness Perfect FGM); CT12 + 10 CP (Colgate Total® 12); ES + 10 CP (Elmex® Sensitive); BPC + 10 CP (Bianco® ProClinical); CMP + 10 CP (Colgate® Máxima Proteção Anticáries). The color was evaluated by reflectance spectrophotometry (∆E*ab, ∆E00, and ∆WID) at times T1 (baseline), T2 (24 hours after brushing), and T3 (24 hours after bleaching). Knoop microhardness (KHN) analysis were performed at T3. The enamel surface was qualitatively analyzed by Scanning Electron Microscopy (SEM). The data were analyzed using generalized linear models through descriptive and exploratory analyses, and a significance level of 5% was considered.

**Results:**

Significant differences were observed when the bleached groups were compared to the NC group for ∆E*ab, ∆E00, and ∆WID at time T3 (*p*= <0.0001). However, the bleached groups presented no significant differences regarding studied times (*p*> 0.05). KHN did not differ significantly among the six groups (*p*=0.7585).

**Conclusions:**

Toothpastes with tricalcium phosphate (β-TCP) do not intervene with the efficacy of bleaching treatment with 10% carbamide peroxide. Although a slight mineral deposition on enamel surface can be observed on SEM images, KHN was not significantly altered, and the polishing of the samples were maintained.

** Key words:**Dental Bleaching, Carbamide Peroxide, Hydrogen Peroxide, Dental Enamel, Tricalcium Phosphate.

## Introduction

At-home bleaching using low-concentration gels, preferably 10% carbamide peroxide (10 CP), is a highly recommended bleaching technique due to its easy application and reduced chair time and costs (1,2). While these gels have CP as an active component, hydrogen peroxide (HP) is the active factor responsible for teeth whitening (3). Their whitening mechanism comprises the dissociation of the 10 CP into HP, corresponds to one-third of the CP percentage (3,35% HP), and urea (6,65%) (1).

Due to its low molecular weight, HP penetrates mineralized tooth tissues by diffusion (4). Then, it dissociates into water and oxygen radicals, which react with organic chromogens at dentin (5), promoting their breakdown by a redox reaction (6). The breakdown of the organic chromogens decreases light absorption and allows them to be removed from the tooth structure, resulting in whiter and less yellow teeth (7).

However, many studies have shown adverse effects when using these oxidative agents, such as calcium and phosphate mineral loss (8) resulting in changes in the enamel’s physical and topographical properties (9). These deleterious effects are caused by HP (8) and can be reversed through the application of remineralizing agents, in charge of calcium and phosphate ions deposition on the enamel surface (10-12).

In this context, toothpastes have been improved to help minimize problems affecting the oral mucosa and dental elements (13). Therefore, dental treatments are being performed in association with several active ingredients incorporated in different toothpastes that have possible remineralizing potential in the face of erosive challenges (14,15). Tricalcium phosphate (β-TCP) is an active factor that has recently been introduced (16). It acts as a remineralizing source (17) since it dissociates into Ca2+ and PO43+ ions, essential for dental remineralization, making them available in the environment as single ions or their agglomerates (18). In addition, this active ingredient has reduced solubility when compared to other calcium and mineral salts, allowing it to act as a source of mineral components (19).

Due to the modern population’s lifestyle, more acidic diet, and constant search for a more harmonious aesthetic (20-22), studies on remineralizing agents are fundamental (23). Consequently, it is important to evaluate the remineralizing potential of toothpastes containing the β-TCP particles in the context of supervised home bleaching with 10 CP, since there are no *in vitro* studies on their effects on bleaching techniques with low-concentration gels.

Therefore, this study aimed to evaluate the effect of two commercial toothpastes containing β-TCP in their formulation, when applied before bleaching with 10 CP, and the capability of promoting changes in dental enamel color and microhardness, to determine whether it contributes to minimizing the deleterious effects of low-concentration bleaching gels based on 10 CP. The null hypotheses tested were: (1) The evaluated commercial toothpastes would not change the teeth color after brushing followed by whitening with 10 CP; (2) Toothpastes containing β-TCP would not change the microhardness of dental enamel after brushing followed by bleaching with 10 CP; (3) The dental enamel’s superficial topography would not be altered after brushing with toothpastes containing β-TCP followed by bleaching with 10 CP.

## Material and Methods

-Experimental Design

**Figure 1:**
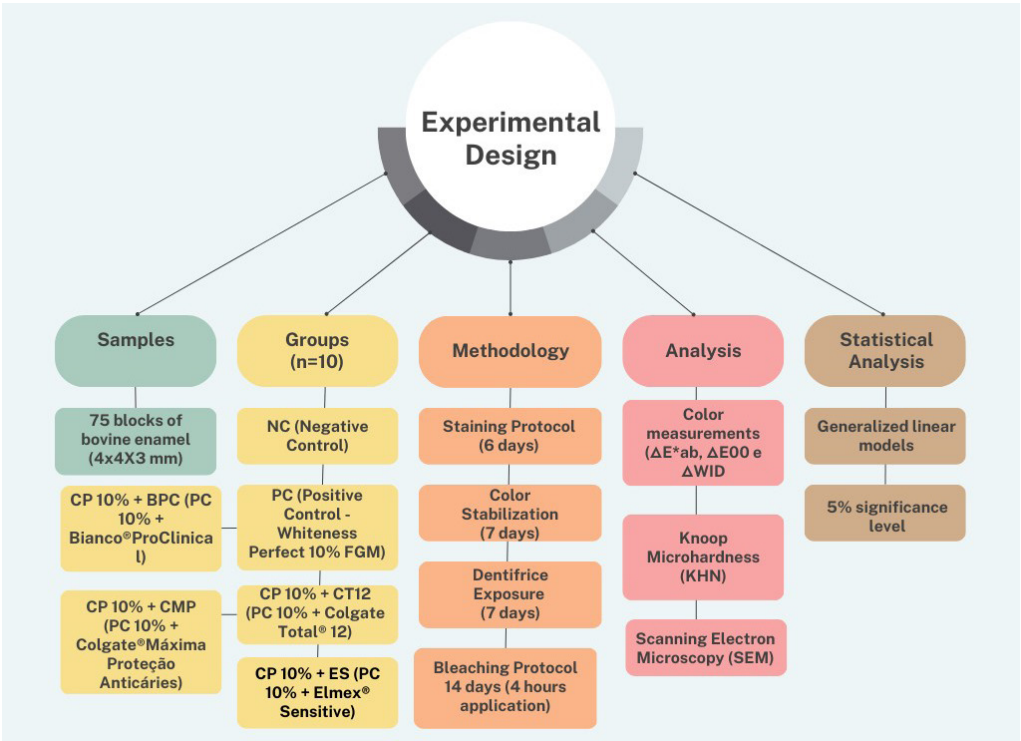
Experimental design of the study.

Sample calculation was calculated with the G*Power program (n=10) with a large effect size (f=0.38) and power of at least ≥80% (β=0.20) for the main effects: group and time, considering the interaction (group × time). The significance level was 5% (α=0.05). The experimental design of the study is presented in Figure 1.

-Sample Preparation

The samples were obtained from bovine incisors (24), which were stored in a 0.1% thymol solution (Proderma, *Pi*racicaba, São Paulo, Brazil). Then, the teeth were cleaned with a scalpel, and polished using rubber cups (KG Sorensen, Ind. Com. Ltda, Barueri, SP, Brazil) and pumice paste (SS White LTDA; Rio de Janeiro, RJ, Brazil) and water (2:1). The crown was separated from the root with a double-sided diamond disk (KG Sorensen, Ind. Com. Ltda., Barueri, SP, Brazil) under irrigation in a low-speed micromotor (Dabi Atlante; Ribeirão Preto, SP, Brazil). Next, samples were cut from the coronary portion in the mesiodistal and incisocervical directions using a metallographic cutter (Isomet 1000, Buehler) and a high-precision diamond disk (4”×012×½, Buehler, Illinois, USA). The samples were cut into the dimensions of 16 mm2 (4 × 4 mm- 1mm enamel and 2mm dentin). Then, the dentin and enamel surfaces were flattened and polished with silicon carbide sandpapers in crescent granulation (600-grit, 1200-grit, 2500-grit, 4000-grit) under constant irrigation using a rotary polisher (Arotec, Cotia, SP, Brazil). Polishing was performed using felts discs and metallographic diamond pastes of decreasing granulation (1 µm, ¼ µm). Finally, the samples were placed in an ultrasonic machine (Marconi Piracicaba, São Paulo – Brazil) for 15 minutes to remove any debris from the surface.

-Staining Protocol

Each sample lateral surface was protected with a colorless resistant acid varnish (Risqué Colorless, Taboão da Serra, SP, Brazil) to protect the dentin during the staining protocol, permitting the contact of the staining solution and bleaching gel with the enamel only (25).

A solution of black tea (1.8g) (Dr. Oetker LTDA, São Paulo, SP, Brazil) and 100mL of distilled water was daily prepared. The solution was heated for three minutes. Next, samples were infused in boiling water for five minutes. Then, they were submerged in the solution and stored in an oven (at 37 ± 1ºC) for six days, with the solution renewal every 24 hours. Samples were stored in artificial saliva (pH 7.0; composition: Ca 1.5 mmol/L; *P* 0.9 mmol/L; KCl mmol/L; 0.1mol/L tris buffer) at 37 ± 1ºC for seven days. To achieve color stabilization (7,8,25,26) this solution was replaced daily (7,8,25,26). Finally, prophylaxis was performed on enamel and dentin surfaces using a 2:1 mixture of pumice and water (25). To return the polished and smooth surface of the enamel after the staining protocol, it is preferable a final polishing with #4000 silicon carbide sandpaper (SiC).

-Sample Allocation, randomization, and blinding of the study

To assess whether the initial sample was homogeneous and correctly distribute the blocks, based on these data in different groups, the criteria for selecting the samples were based on the mean and standard deviation of the surface microhardness (KHN). Samples with a standard deviation higher (intra-block variability) or lower than 10% of their mean values for individual microhardness were excluded from the study (inter-block variability) (27).

Samples were randomized (n=10) into groups according to the bleaching protocol and toothpastes application. The randomization was made after initial color analysis, considering the initial values for L* parameter of each sample, which was used to stratify and allocate specimens in all groups, to reduce the initial variability between groups tested (28). Toothpastes was removed from their original packaging and transferred into another vial. A second researcher performed this procedure to ensure blinding of the study, allowing no influence on the knowledge of the treatment being performed in each group.

-Toothpaste Exposure

Samples were randomly divided into six groups considering toothpaste and bleaching protocols (n=10): (NC) Negative Control (no brushing or bleaching), with artificial saliva daily replacement; (10 CP) (Positive Control- bleaching with 10% CP); (CT12 + 10 CP) exposed to Colgate Total 12 (CT12) toothpaste then 10% CP; (ES + 10 CP) exposed to Elmex Sensitive (ES) toothpaste then 10% CP; (BPC + 10 CP) exposed to Bianco Pro Clinical (BPC) toothpaste then 10% CP; (CMP + 10 CP) exposed to Colgate Máxima Proteção Anti-Cáries (CMP) toothpaste then 10% CP. Chart 1 shows in detail the technical profile of the toothpastes used. A solution consisting of toothpaste and distilled water (25), was prepared forming a “slurry” solution (1:3) (25).

An electric toothbrush (Oral-B Professional Care 3000; Oral-B, Schwalbach am Taunus, Germany) (29) was chosen to perform the brushing protocol. To maintain the standardization of toothbrush position, a custom dense silicone (Express™ XT Denso - 3M) device was used, allowing the parallelism of the toothbrush and the enamel surface (30). Pressure exerted on the sample surface was normalized at 2.5 N, indicated by a light alert. The toothbrush was triggered for 15 seconds on each surface (31). After this procedure, samples were kept in the “slurry” solution for 2 minutes. Then, they were cautiously washed with distilled water and stored in artificial saliva. The brushing protocol was performed daily for seven days (28,32). Toothbrushing was performed before the bleaching treatment as reported in previous studies (24,32). (Supplement 1) (http://www.medicinaoral.com/medoralfree01/aop/jced_61300_s01.pdf)

-Bleaching Protocol

Samples were submitted to bleaching treatment with a 10% CP gel (Whiteness Perfect, FGM, Joinville – Santa Catarina/ Brazil) for 14 days, following at-home bleaching technique, with daily application of 4 hours. Subsequently, the gel was removed cautiously with a cotton swab, and the samples were washed abundantly with distilled water to remove remnants of bleaching gel. After each session, the samples were stored in artificial saliva, with daily exchanges.

-Color Measurements

To analyze color measurement a calibrated spectrophotometer (Konica Minolta CM- 700d) was chosen. The readings were performed in triplicate and the average for each coordinate was recorded. All samples were placed in a Teflon compartment inside a light chamber (GTI Newburg, NY, USA), using the “daylight” mode for standardization. Parameters of the CIELAB (∆E*ab), CIEDE 2000 (∆E00), and Whiteness Index for Dentistry (∆WID) (33,34) were evaluated, according to the Commission Internationale de l’Eclairage 1978 (CIE; International Commission on Illumination) (35), considering three-dimensional modes L*, a*, and b*.

**Figure 2:**
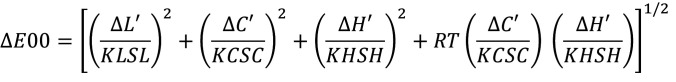
Formula

Parameter L* characterizes luminosity from black to white (0 -100), a* consider the color variation along the red-green axis, and b* is the measurement along the yellow-blue axis (36,37). To calculate CIELAB, the formula ΔE*ab= [(L1 - L0)2 + (a1 - a0)2 + (b1 - b0)2]1/2, was applied. For CIEDE 2000, the equation considered was, (Fig. 2):

According to the equations, ΔL*, Δa*, and Δb* characterizes the differences among the times for the values L, a and b, respectively. ΔL’, ΔC’, and ΔH’ represents the differences in value (light), chroma, and hue, correspondingly. SL, SC, and SH adjust coordinate values as a function of variation in color difference. KL, KC, and KH are parameters for corrections relative to experimental conditions, while RT consider the interaction between chroma and hue differences in the blue region (33,34). To calculate Whiteness Index for Dentistry (ΔWID), the following formula was selected: WID=0,511L*−2,324a*−1,100b* (38). For all calculations limits of perceptibility and acceptability were considered (38): 50%:50% perceptibility (1.22 ΔE*ab; 0.81 ΔE00; 0.61 ΔWID); 50%:50% acceptability (2.66 ΔE*ab; 1.77 ΔE00; 2.90 ΔWID).

Evaluations were performed three times: before treatment (T1), 24 hours after exposure to toothpaste (T2), and 24 hours after bleaching treatment (T3).

-Surface Microhardness (KHN)

Microhardness analyses were performed with Shimadzu HMV-2000 (Tokyo, Japan). The specimens were positioned with the enamel surface parallel to the Knoop indentation. Then, five indentations were made in the center of each sample, considering a load of 50g, the time of 5 seconds, and a distance of 100µm between each indentation. The arithmetic mean was measured by means of 5 indentations. Enamel indentations can change the topography of the surface, thus, modifying its light reflectance pattern, with negative impact on color analysis. Therefore, KHN was determined 24 hours after bleaching treatment (T3). The bleached groups results were compared with the NC group (32).

-Scanning Electron Microscopy (SEM)

Three samples were randomly chosen at the end of the study for qualitative analysis of the enamel. A scanning electron microscope (Jeol, JSM 5600LV, Tokyo, Japan) was used for topographic analysis of the enamel surface at 15 kV and 4000× magnification. Each sample was previously metalized (Bal-Tex SCD 050 sputter coter, Germany) with gold alloy.

-Statistical Analysis

The data were submitted to homogeneity of variance by the Levene test, and normality test using Shapiro–Wilk. Next, descriptive, and exploratory analyses of the data were performed. Next, data were analyzed using generalized linear models for color and surface microhardness with R* statistical software, considering a 5% significance level.

*R Core Team (2022). R: A language and environment for statistical computing. R Foundation for Statistical Computing, Vienna, Austria.

## Results

-Color (∆E*ab; ∆E00)

• 24 hours after toothbrushing 

∆E*ab and ∆E00 did not differ significantly between groups at time T2 compared to T1 (*p*=0.1925; *p*=0.3923, respectively) (Table 1).

**Table 1:**
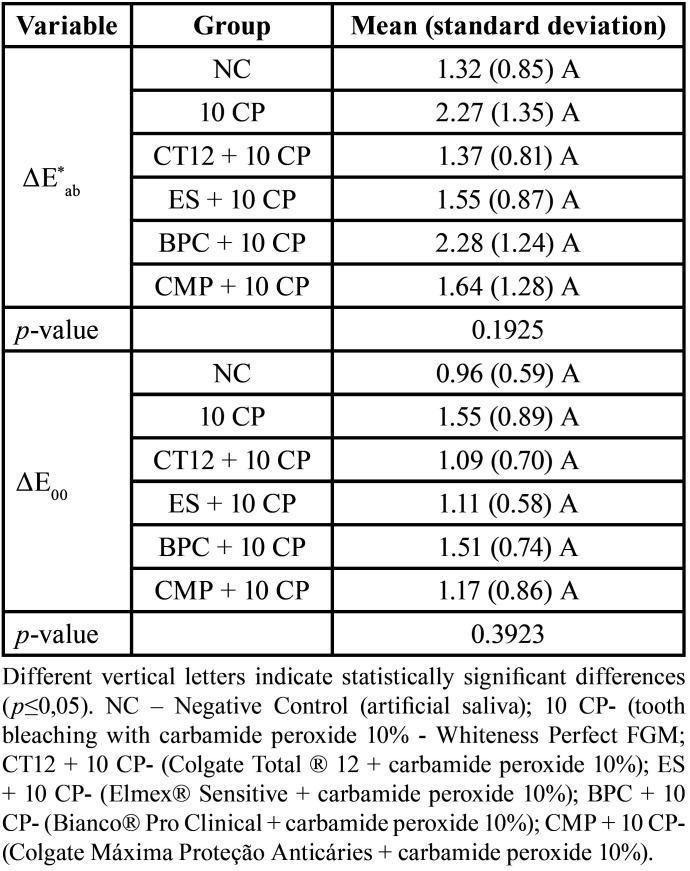
Mean (standard deviation) of color variants, ΔE*ab and ΔE00, according to the groups, 24 hours after brushing (T2).

• 24 hours after bleaching

∆E*ab and ∆E00 values were significantly lower in the NC group than in the bleaching groups at time T3 compared to times T1 and T2 (*p*<0.0001). ∆E*ab and ∆E00 did not differ significantly among the five bleaching groups (*p* > 0.05) (Table 2).

**Table 2:**
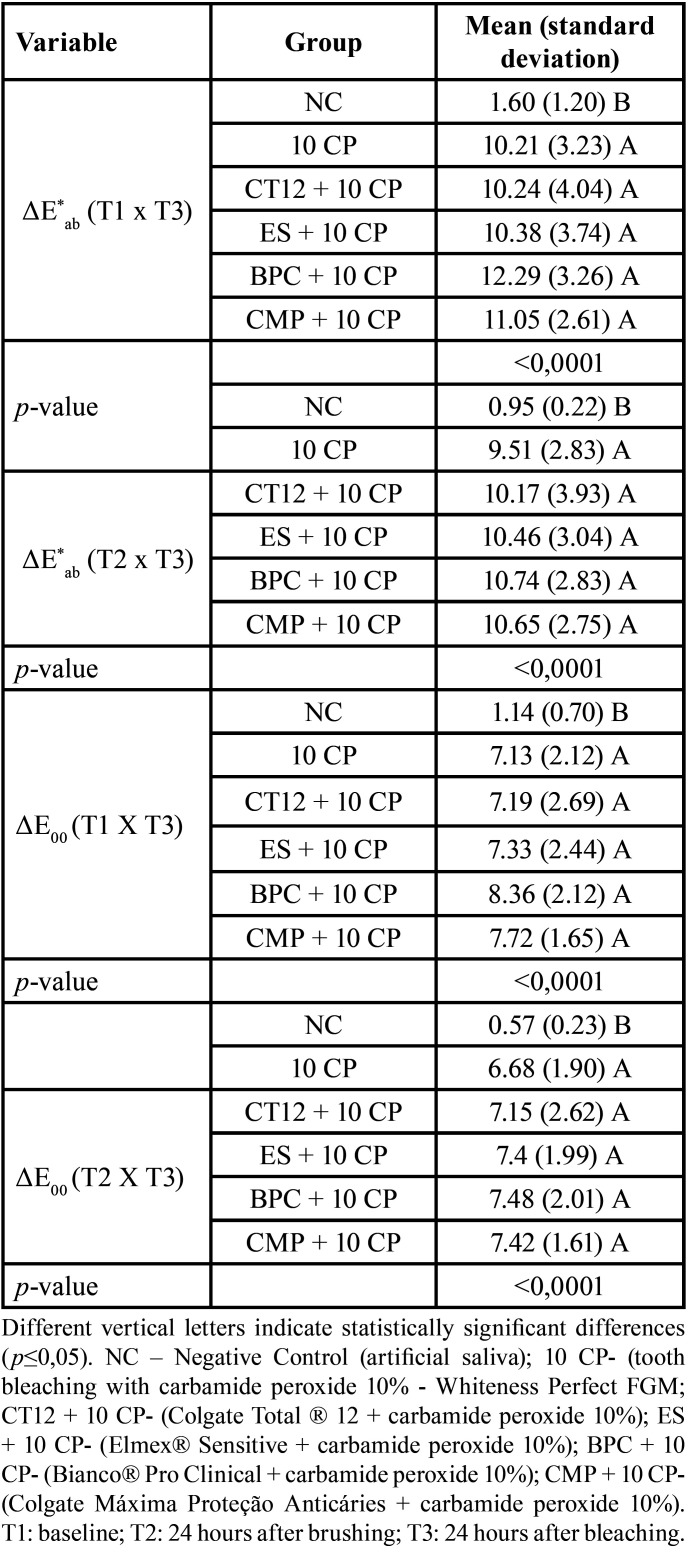
Mean (standard deviation) of color variants, ΔE*ab, and ΔE00, according to the groups, 24 hours after bleaching (T3).

• Whiteness Index for Dentistry (∆WID)

∆WID did not differ significantly between groups at times T1 and T2 (*p*=0.2637; *p*=0.5197, respectively). ∆WID in T3 was significantly lower in the NC than in the other groups (*p*<0.0001). ∆WID did not differ significantly among the five bleaching groups (*p*>0.05) (Table 3).

**Table 3:**
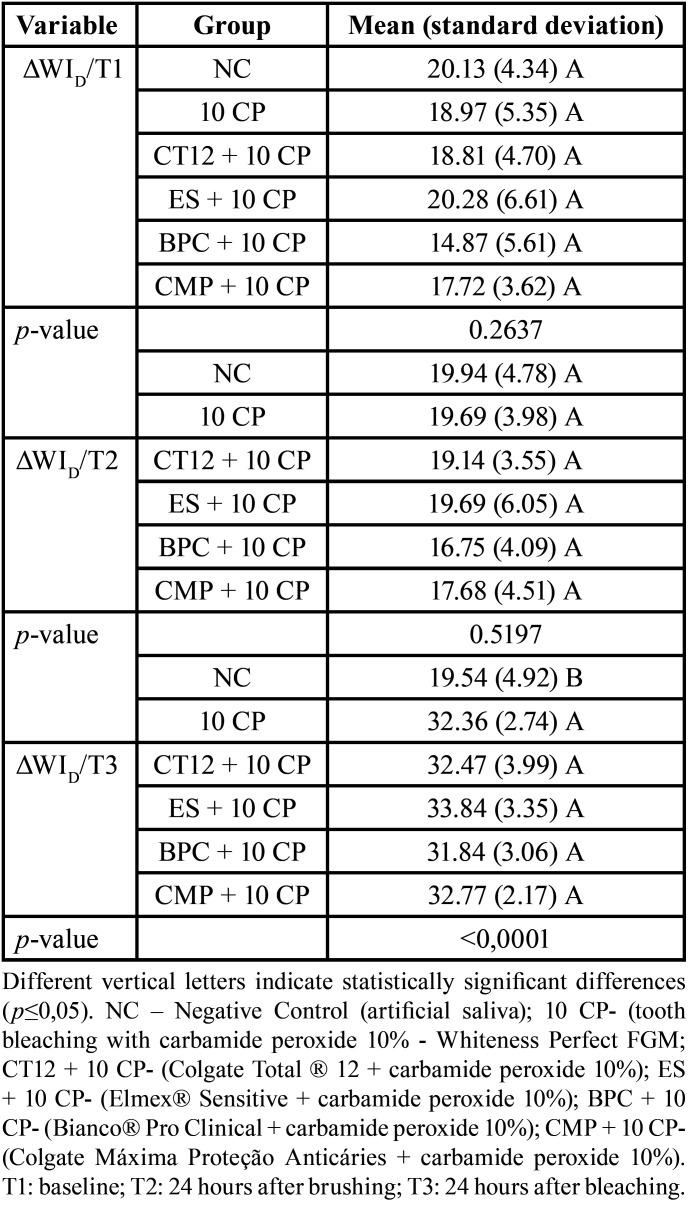
Mean (standard deviation) of ΔWID, according to the groups, in the times T1, T2 and T3.

-Knoop Microhardness (KHN)

Table 4 shows the results of KHN. It can be observed that there was no significant difference in KHN values considering the comparison between NC and the groups bleached with CP 10 (*p*=0.7585).

**Table 4:**
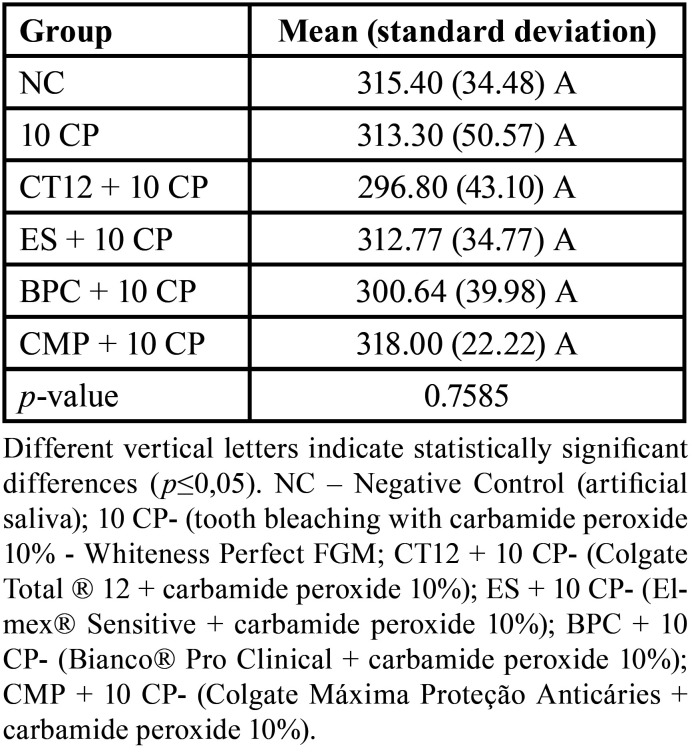
Mean (standard derivation) of microhardness (KHN) according to the group.

-Scanning Electron Microscopy (SEM)

**Figure 3:**
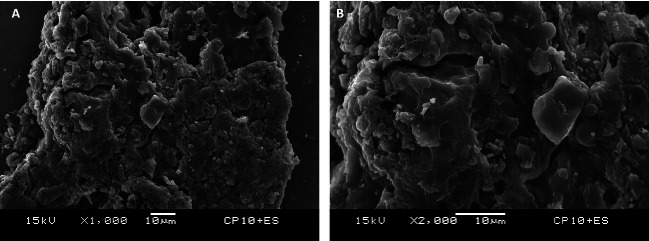
Low (1000x) (A) and (B) high magnification (2000x) SEM view of the β-tricalcium phosphate particles present on commercial toothpaste Elmex Sensitive (ES). β-tricalcium phosphate particles are characterized by the presence of agglomerated mineralized particles with different morphology of various irregular sized polygonal crystals that ranges from 500 nm to 1500 nm.

**Figure 4:**
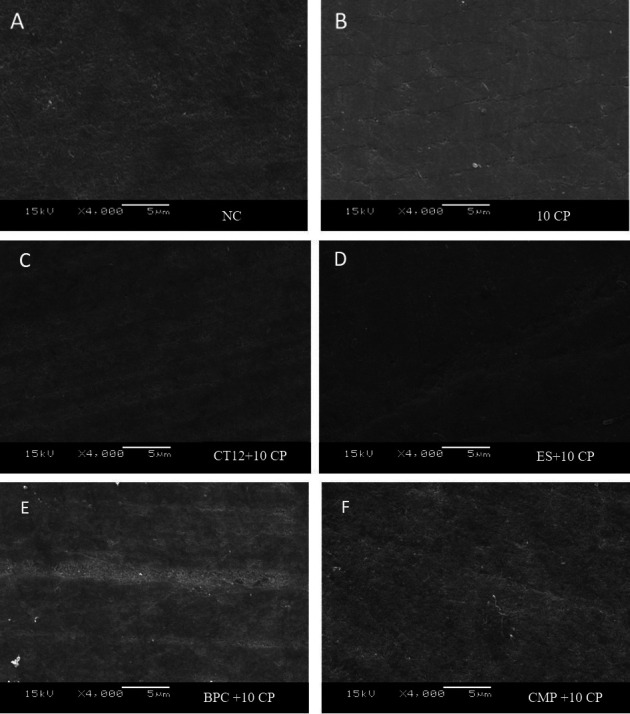
A. NC (negative control); B. 10 CP (Positive control with CP 10% Whiteness Perfect FGM); C. CT12 + 10 CP (CP 10% + Colgate Total ® 12); D. ES + 10 CP (CP 10% + Elmex® Sensitive); E. BPC + 10 CP (CP 10% +Bianco® Pro Clinical); F. CMP + 10 CP (CP 10% + Colgate Máxima Proteção Anticáries).

β-tricalcium phosphate particles morphology is demonstrated in Figure 3. It can be observed the presence of agglomerated mineralized particles with different sizes ranging from 500 nm to 1500 nm. The enamel in the NC group present polished and smooth appearance (Fig. 4A). The groups that were submitted to bleaching treatment present slight demineralization of the aprismatic enamel layer (Fig. 4B-F). Additionally, the groups treated with toothpastes containing β-TCP shows the presence of whitish spots indicative of mineral deposition in enamel. However, in all images it is observed that the polishing and smoothness of the enamel was properly maintained, similarly to the NC group (Fig. 4A).

## Discussion

The first null hypothesis was accepted since there was no significant color alteration after brushing protocol with toothpastes containing β-TCP associated with 10 CP. Extrinsic and intrinsic staining factors determine tooth color (6,21). Extrinsic staining corresponds to pigmentation depositions on the surface of the acquired pellicle from food and habits, such as smoking (21). These pigments are easily removed by brushing with toothpaste. While brushing contributes to a more aesthetic and less yellowish appearance (6,22), interference with the intrinsic tooth color factors is necessary to achieve a significant color alteration (7). Intrinsic staining is caused by chromogen molecules inside the dentin that absorb light and reflect the color of the tooth surface, due to enamel’s translucency (22).

Therefore, to promote an effective color alteration, it is necessary to perform bleaching procedures, with CP or HP, in high (in-office bleaching) or low (supervised at-home bleaching) concentrations (3). In this study, groups that underwent bleaching with 10 CP showed color changes when compared to the NC group. Due to its low molecular weight, hydrogen peroxide penetrates the tooth’s mineralized structures (4) and breaks down the chromogenic molecules in the dentin through a redox reaction (5). Breaking down and removing these molecules from tooth structure decreases light absorption, resulting in whiter teeth (7).

ΔE*ab and ΔE00 differed significantly in all groups when compared to NC group. Additionally, all groups treated with 10 CP presented statistical similarities. It is possible to conclude that the 14-day, with daily four-hour applications using 10% CP in association with prior brushing with toothpaste containing β-TCP, did not significantly affected the efficacy of the bleaching treatment.

Regarding to the whiteness index (∆WID), it was observed that the groups submitted to 10 CP treatment only differed from the NC group in T3 (24 hours after bleaching), which was previously expected, because the exposure only to the toothpaste is not able to cause color alteration, since the toothpastes used in this study do not present bleaching agents in the formulation such as CP and HP.

The second null hypothesis was accepted since there were no significant differences in KHN after brushing followed by bleaching with 10 CP. Tooth bleaching is considered a safe and effective technique when correctly performed (20). However, some studies have reported changes to the enamel surface, such as alterations in enamel´s microhardness and topography due to mineral content loss (8,9).

Nevertheless, this study did not identify significant changes in KHN after a bleaching protocol with 10 CP. Lopes *et al*. 2022 (11) found significant changes in enamel microhardness when brushing was performed with the same toothpastes evaluated in this study. Though, in this previous study the bleaching protocol evaluated used a higher concentration (Whiteness HP 35% FGM, SP, Brazil). This study aimed to compare whether similar results would be found when using lower concentration bleaching gel (10 CP). This study show that 10 CP does not significantly alter the enamel microhardness, in agreement with previous studies that showed no significant changes in the dental enamel surface microhardness and roughness (2).

Some factors may have contributed to the maintenance of enamel microhardness. First, the samples were kept in artificial saliva solution throughout the 14 days of experiment, with daily replacement, simulating an oral clinical situation. The artificial saliva solution used in this study (32) is a remineralizing solution, which favors mineral deposition on the enamel surface. Secondly, all toothpastes analyzed in this study contained fluoride in its composition. Fluoride is a remineralizing agent commonly used in commercial toothpastes (2,12). In agreement with Juntavee *et al*. 2021(12), which emphasizes the capability of mineral recovery provided by fluoride ions present in human saliva, and the use of fluoridated solutions. Finally, the lower bleaching agent concentration (10%) should be considered since a previous study with a higher bleaching agent concentration (35%) reported higher microhardness values (11).

In addition to compounds containing fluoride as remineralizing agents (32), particles such as β-TCP (17) have recently been introduced into toothpastes formulations, with the objective to minimize the deleterious effects promoted by bleaching agents on enamel (32). β-TCP composition is based on calcium and phosphate ions that act as a source of mineral components (19). Their mechanism of action occurs when in contact with the teeth moistened surface, breaking the protective barrier, and releasing calcium, phosphate, and fluoride ions, which become available in the oral environment for deposition on the dental enamel surface (12).

Therefore, the maintenance of enamel microhardness may reflect the combined effects of the artificial saliva remineralizing effects and the use of toothpastes containing β-TCP, fluoride and a low-concentration bleaching gel (10 CP). Juntavee *et al*. 2021 (12) discussed that remineralization occurs as a dynamic process, to maintain a balance in the teeth hard tissues.

The third null hypothesis was accepted since there were no significant alterations in the dental enamel surface topography regarding polishing and smoothness. Slight mineral deposition was detected on the surface of samples brushed with toothpastes containing β-TCP, represented by whitish spots on the enamel surface. This mineral deposition can be explained by the presence of remineralizing particles on toothpaste formulation (β-TCP and fluoride). However, further studies should be conducted to confirm the presence of this particle on the sample’s surfaces.

Recently, de Amoêdo Campos Velo *et al*. 2020 (16), showed β-TCP on the toothpastes can be interesting to repair active dentin caries lesions. According to the authors, in a long-term process, the continuous using of such toothpastes can be an interesting option to patients at high-risk, such as the elderly population or patients under local/systemic alterations resulting in low salivary flow or compromised quality of saliva, especially when associated with fluoride in the composition, as well as the toothpastes analyzed in the present study.

In addition, as previously discussed, saliva is fundamentally important in the process of dental remineralization. Daily replacement of this solution may have contributed to maintaining the teeth mineral content. Nevertheless, to verify these results in more reliable conditions, further studies are indicated, to evaluate the inorganic content of dental enamel.

## Conclusions

Given the clinical limitations of this study, its findings support the following conclusions:

1. Brushing with commercial toothpaste containing β-TCP before bleaching does not interfere with the bleaching efficacy of 10% CP;

2. Brushing with commercial toothpaste containing β-TCP before bleaching with 10% CP does not change the dental enamel microhardness;

3. Commercial toothpaste with β-TCP do not cause significant changes in enamel surface topography regarding polishing and smoothness.

## References

[1] Luque-Martinez I, Reis A, Schroeder M, Muñoz MA, Loguercio AD, Masterson D (2016). Comparison of efficacy of tray-delivered carbamide and hydrogen peroxide for at-home bleaching: a systematic review and meta-analysis. Clin Oral Investig.

[2] Zanolla J, Marques ABC, Costa DC, Souza AS, Coutinho M (2017). Influence of tooth bleaching on dental enamel microhardness: a systematic review and meta-analysis. Aust Dent J.

[3] Kwon SR, Wertz PW (2015). Review of the Mechanism of Tooth Whitening. J Esthet Restor Dent.

[4] Eimar H, Ghadimi E, Marelli B, Vali H, Nazhat SN, Amin WM (2012). Regulation of enamel hardness by its crystallographic dimensions. Acta Biomaterials.

[5] Ontiveros JC, Paravina RD (2009). Color change of vital teeth exposed to bleaching performed with and without light. J Dent.

[6] Côrtes G, Pini NP, Lima DA, Liporoni PC, Munin E, Ambrosano GM (2013). Influence of coffee and red wine on tooth color during and after bleaching. Acta Odontol Scand.

[7] Sulieman M (2004). An overview of bleaching techniques: I. History, chemistry, safety and legal aspects. Dent Update.

[8] Zeczkowski M, Tenuta LMA, Ambrosano GMB, Aguiar FHB, Lima DANL (2015). Effect of different storage conditions on the physical properties of bleached enamel: An in vitro vs. in situ study. J Dent.

[9] D'Amario M, D'Attilio M, Baldi M, De Angelis F, Marzo G, Vadini M (2012). Histomorphologic alterations of human enamel after repeated applications of a bleaching agent. Int J Immunopathol Pharmacol.

[10] Coseska E, Gjorgievska E, Coleman NJ, Gabric D, Slipper IJ, Stevanovic M (2016). Enamel alteration following tooth bleaching and remineralization. Jounal of Microscopy.

[11] Lopes MP, Gonçalves IMC, Garcia RM, Sobral-Souza DF, Aguiar FHB, Lima DANL (2022). Influence of toothpastes containing tricalcium phosphate on dental enamel microhardness, color, and topography. Research, Society and Development.

[12] Juntavee A, Juntavee N, Hirunmoon P (2021). Remineralization Potential of Nanohydroxyapatite Toothpaste Compared with Tricalcium Phosphate and Fluoride Toothpaste on Artificial Carious Lesions. Int J Dent.

[13] Ganss C, Schulze K, Schlueter N (2013). Toothpaste and erosion. Monogr Oral Sci.

[14] Korner P, Inauen DS, Attin T, Wegehaupt FJ (2020). Erosive/Abrasive enamel wear while using a combination of anti-erosive toothbrush/paste. Oral Health Prev Dent.

[15] Tomaz PLS, Sousa LA, Aguiar KF, Oliveira TS, Matochek MHM, Polassi MR (2020). Effect of 1450-ppm fluoride containing toothpastes associated with booters on the enamel remineralization and surface roughness after cariogenic challenge. Eur J Dent.

[16] de Amoêdo Campos Velo MM, Agulhari MAS, Rios D, Magalhães AC, Honório HM, Wang L (2020). Root caries lesions inhibition and repair using commercial high-fluoride toothpastes with or without tri-calcium phosphate and conventional toothpastes containing or not 1.5% arginine CaCO3: an in situ investigation. Clin Oral Investig.

[17] Viana ÍEL, Lopes RM, Silva FRO, Lima NB, Aranha ACC, Feitosa S (2020). Novel fluoride and stannous -functionalized β-tricalcium phosphate nanoparticles for the management of dental erosion. J Dent.

[18] Jin J, Xu X, Lai G, Kunzelmann KH (2013). Efficacy of tooth whitening with different calcium phosphate-based formulations. Eur J Oral Sci.

[19] Scaramucci T, Borges AB, Lippert F, Frank NE, Hara AT (2013). Sodium fluoride effect on erosion-abrasion under hyposalivatory simulating conditions. Arch. Oral Biol.

[20] Corcodel N, Hassel AJ, Sen S, Saure D, Rammelsberg P, Lux CJ (2017). Effect of enamel sealants on tooth bleaching and on the color stability of the result. Odontology.

[21] Lee WF, Takahashi H, Huang SY, Zhang JZ, Teng NC, Peng PW (2022). Effects of At-Home and In-Office Bleaching Agents on the Color Recovery of Esthetic CAD-CAM Restorations after Red Wine Immersion. Polymers.

[22] Joiner A, Luo W (2017). Tooth colour and whiteness: A review. J Dent.

[23] Kanzow P, Wegehaupt FJ, Attin T, Wiegand A (2016). Etiology and pathogenesis of dental erosion. Quintessence Int.

[24] Zamudio-Santiago J, Ladera-Castañeda M, Santander-Rengifo F, López-Gurreonero C, Cornejo-Pinto A, Echavarría-Gálvez A (2022). Effect of 16% Carbamide Peroxide and Activated-Charcoal-Based Whitening Toothpaste on Enamel Surface Roughness in Bovine Teeth: An In Vitro Study. Biomedicines.

[25] Lima DA, Silva AL, Aguiar FH, Liporoni PC, Munin E, Ambrosano GM (2008). In vitro assessment of the effectiveness of whitening dentifrices for the removal of extrinsic tooth stains. Braz Oral Res.

[26] Serra MC, Cury JA (1992). The in vitro effect of glass-ionomer cement restoration on enamel subjected to a demineralization and remineralization model. Quintessence Int.

[27] Pin WF, Benati MRL, Souza AGC, Ferraz LN, Vitti RP, Scatolin RS (2022). Evaluation of mineral content of tooth enamel after application violet led associated with 35% hydrogen peroxide. Photodiagnosis Photodyn Ther.

[28] Vieira-Junior WF, Ferraz LN, Giorgi M, Ambrosano G, Aguiar F, Lima D (2019). Effect of Mouth Rinse Treatments on Bleached Enamel Properties, Surface Morphology, and Tooth Color. Oper Dent.

[29] Schulueter N, Klimek J, Ganss C (2014). Effect of a chitosan additive to a Sn2+ -containing toothpaste on its anti- erosive/anti-abrasive efficacy - a controlled randomized in situ trial. Clin Oral Invest.

[30] Moron BM, Miyazaki SS, Ito N, Wiegand A, Vilhena F, Buzalaf MA (2013). Impact of different fluoride concentrations and pH of dentifrices on tooth erosion/abrasion in vitro. Aust Dent J.

[31] Comar LP, Gomes MF, Ito N, Salomão PA, Grizzo LT, Magalhães AC (2012). Effect of NaF, SnF(2), and TiF(4) Toothpastes on Bovine Enamel and Dentin Erosion-Abrasion In Vitro. Int J Dent.

[32] Vieira-Junior WF, Lima DANL, Tabchoury CPM, Ambrosano GMB, Aguiar FHB, Lovadino JR (2016). Effect of Toothpaste Application Prior to Dental Bleaching on Whitening Effectiveness and Enamel Properties. Oper Dent.

[33] Pérez MM, Pecho OE, Ghinea R, Pulgar R, Bona AD (2019). Recents advances in Color and Whiteness Evaluations in Dentistry. Current Dentistry.

[34] Ramos NC, Luz JN, Valera MC, Melo RM, Saavedra GSFA, Bresciani E (2019). Color Stability of Resin Cements Exposed to Aging. Oper Dent.

[35] Públio JC, Zeczkowski M, Burga-Sánchez J, Ambrosano GMB, Groppo FC, Aguiar FHB (2019). Influence of different thickeners in at-home tooth bleaching: a randomized clinical trial study. Clin Oral Investig.

[36] Pan Q, Westland S (2018). Tooth color and whitening - digital technologies. J Dent.

[37] Gómez-Polo C, Muñoz MP, Luengo MCL, Vicente P, Galindo P, Casado AMM (2016). Comparison of the CIELab and CIEDE2000 color difference formulas. J Prosthet Dent.

[38] Pecho OE, Martos J, Pinto KVA, Pinto KVA, Baldissera RA (2019). Effect of hydrogen peroxide on color and whiteness of resin-based composites. J Esthet Restor Dent.

